# Supplementary material for: Conformational change of Dishevelled plays a key regulatory role in the Wnt signaling pathways
Source: eLife. 2015 Aug 22;4:e08142. doi: 10.7554/eLife.08142 (PMC4577825; doi:10.7554/eLife.08142)
Supplement: Figure 5—source data 1. — DOI: http://dx.doi.org/10.7554/eLife.08142.008 [file elife08142s001.docx]

**Figure 5-source data 1.** Intermolecular NOEs between the Dvl-C peptide and the PDZ domain obtained from ^13^C-half-filtered NOESY-HSQC spectra^a^.

| Assignment | ω_1_ | ω_2_ | ω_3_ | Intensity |
| --- | --- | --- | --- | --- |
| F3HB3-I266CG2-QG2 | 2.755 | 15.898 | 0.537 | w |
| F3HZ-I266CD1-QD1 | 7.049 | 10.537 | 0.494 | w |
| F3HZ-I266CG2-QG2 | 7.066 | 15.889 | 0.542 | w |
| V5QG1-I266CD1-QD1 | 0.706 | 10.603 | 0.496 | s |
| V5QG1-I266CG2-QG2 | 0.709 | 15.828 | 0.539 | s |
| V5QG1-L321CD1-QD1 | 0.708 | 22.460 | 0.534 | w |
| V5QG1-L321CD2-QD2 | 0.708 | 21.932 | 0.605 | w |
| V5QG2-I266CD1-QD1 | 0.712 | 10.564 | 0.440 | m |
| V5QG2-I266CG2-QG2 | 0.715 | 15.835 | 0.548 | s |
| V5QG2-V318CG1-QG1 | 0.716 | 19.883 | 0.917 | m |
| D6HA-I266CG2-QG2 | 4.388 | 15.760 | 0.548 | w |
| M8H-I264CD1-QD1 | 7.866 | 10.407 | 0.443 | s |
| M8QE-I264CD1-QD1 | 1.852 | 10.472 | 0.444 | m |
| M8QE-I266CD1-QD1 | 1.866 | 10.460 | 0.49 | m |
| M8QE-V318CG1-QG1 | 1.856 | 19.950 | 0.916 | s |
| M8QE-L321CD1-QD1 | 1.870 | 22.562 | 0.535 | s |
| M8QE-L321CD2-QD2 | 1.867 | 22.053 | 0.609 | w |
| M8QG-I264CD1-QD1 | 2.322 | 10.556 | 0.443 | m |
| M8QG-I266CD1-QD1 | 2.331 | 10.602 | 0.491 | w |
| M8QG-I266CG2-QG2 | 2.316 | 15.760 | 0.548 | w |
| M8QG-V318CG1-QG1 | 2.316 | 20.031 | 0.915 | w |
| M8QG-L321CD1-QD1 | 2.344 | 22.601 | 0.535 | m |

NOE: Nuclear Overhauser effect

^a^ Mixing time, 300 ms.
