# Supplementary material for: Conformational change of Dishevelled plays a key regulatory role in the Wnt signaling pathways
Source: eLife. 2015 Aug 22;4:e08142. doi: 10.7554/eLife.08142 (PMC4577825; doi:10.7554/eLife.08142)
Supplement: Figure 5—source data 2. — DOI: http://dx.doi.org/10.7554/eLife.08142.009 [file elife08142s002.docx]

**Figure 5-source data 2.** Structure statistics for the 15 lowest-energy peptide-PDZ complexes.

| Number of unambiguous constraints |  |
| --- | --- |
| Sequential and medium-range | 25^a^ |
| Intermolecular | 22^b^ |
| Number of ambiguous interaction restraints (AIRs) |  |
| Total AIRs | 48^c^ |
| Intermolecular energies after water refinement |  |
| E_vdw_ (kcal/mol) | -52 ± 2 |
| E_elec_ (kcal/mol) | -186 ± 7 |
| E_noe_ (kcal/mol) | 0.1 ± 0.02 |
| Buried surface area (Å^2^) | 1144 ± 73 |
| Average pairwise RMSD (Å)^d^ |  |
| Heavy | 0.62 ± 0.10 |
| Backbone | 0.37 ± 0.08 |

RMSD: root mean square deviation.

^a^ NOEs from bound peptide.

^b^ NOEs between the Dvl-1 PDZ domain and the Dvl-C peptide.

^c^ Chemical shift perturbation data from NMR titration experiments; solvent accessibility was calculated by using the program NACESS and NOE data.

^d^ Calculated by using the program Molmol.
